# Letter to the Editor: The Association Between Glycemic Variability and the Risk of Diabetic Retinopathy Progression: A Community‐Based Retrospective Cohort Study

**DOI:** 10.1111/1753-0407.70250

**Published:** 2026-07-13

**Authors:** Wenli Xu, Yingyan Ma, Qinping Yang, Qinghua Yan, Jingyan Tian, Yan Shi, Minna Cheng, Lina Lu

**Affiliations:** ^1^ Division of Chronic Non‐Communicable Disease and Injury Shanghai Municipal Center for Disease Control and Prevention Shanghai China; ^2^ Shanghai Eye Diseases Prevention &Treatment Center/Shanghai Eye Hospital, School of Medicine Tongji University Shanghai China; ^3^ Department of Ophthalmology, Shanghai General Hospital, School of Medicine Shanghai Jiao Tong University Shanghai China; ^4^ Eye Public Health Research Center, School of Medicine Tongji University Shanghai China; ^5^ Shanghai Engineering Center for Precise Diagnosis and Treatment of Eye Diseases Shanghai China; ^6^ National Clinical Research Center for Eye Diseases Shanghai China; ^7^ Department of Endocrine and Metabolic Diseases, Shanghai Institute of Endocrine and Metabolic Diseases, Ruijin Hospital Shanghai Jiao Tong University School of Medicine Shanghai China; ^8^ Shanghai National Clinical Research Center for Metabolic Diseases Key Laboratory for Endocrine and Metabolic Diseases of the National Health Commission of the PR China Shanghai China

Dear Editor,

Diabetic retinopathy is among the most clinically significant microvascular complications of diabetes and is a leading cause of preventable blindness [1]. Average glycemic control is an established risk factor [2, 3], but whether glycemic variability contributes independently to retinal damage remains less clear [4, 5]. We examined whether long‐term variability in HbA1c, derived from serial measurements, was associated with diabetic retinopathy progression in a community‐based cohort of patients with type 2 diabetes.

We included 675 adults with type 2 diabetes who underwent fundus examinations in both 2020 and 2023, and who had at least three valid HbA1c measurements during follow‐up. Five variability indices were calculated: coefficient of variation, average real variability, successive variation, variability independent of the mean, and HbA1c variability score. Progression was defined as incident retinopathy, progression to proliferative disease, or worsening within non‐proliferative stages.

Of 675 participants, 125 experienced diabetic retinopathy progression. All variability indices were numerically higher in the progression group, but only HbA1c variability score differed significantly at baseline. After multivariable adjustment, HbA1c variability score remained independently associated with progression; the other indices did not. In stratified analysis, the association was stronger in patients with diabetes duration of 10 years or less but was not significant in those with longer diseases.

These findings suggest that the frequency of clinically meaningful HbA1c fluctuations may matter more than how large individual swings are. The attenuation of the association with longer diabetes duration may reflect the accumulating burden of structural retinal damage, which by then has likely been shaped by years of prior exposure. If replicated, this would argue that the first decade after diagnosis is a particularly important window for glycemic stabilization—not just lowering means HbA1c but reducing repeated excursions.

Practically, HbA1c variability score can be derived from routine clinical measurements, making it a feasible marker for risk stratification without additional testing. Prospective trials are needed to determine whether reducing glycemic instability translates into slower retinopathy progression.

In summary, long‐term HbA1c variability, specifically HbA1c variability score, was independently associated with diabetic retinopathy progression in type 2 diabetes. Glycemic stability, not just glycemic level, may deserve more attention in routine care—particularly early in the disease course.

## Author Contributions

**Wenli Xu:** conceptualization, methodology, investigation, software, formal analysis, writing – original draft, visualization. **Yingyan Ma:** conceptualization, investigation, data curation, writing – review and editing. **Qinping Yang:** conceptualization, methodology, investigation, data curation, writing – review and editing. **Qinghua Yan:** investigation, resources. **Jingyan Tian:** conceptualization, methodology, supervision, writing – review and editing. **Yan Shi:** resources. **Minna Cheng:** supervision, funding acquisition. **Lina Lu:** project administration, supervision.

## Funding

This work was supported by the following: Noncommunicable Chronic Diseases‐National Science and Technology Major Project of China (Grant Nos. 2024ZD0543500 and 2024ZD0524200); Shanghai Municipal Public Health Research Special Project (Grant No. 2024GKM05); Shanghai Municipal Health Commission—Health Industry Youth Project of Clinical Research (Grant No. 20244Y0136); National Natural Science Foundation of China (82370902).

## Conflicts of Interest

The authors declare no conflicts of interest.

## Data Availability

Research data contain personally identifiable information and are not shared.
