# Correction to “Letter to the Editor: The Association Between Glycemic Variability and the Risk of Diabetic Retinopathy Progression: A Community‐Based Retrospective Cohort Study”

**DOI:** 10.1111/1753-0407.70267

**Published:** 2026-11-04

W. Xu , Y. Ma , Q. Yang , et al., “Letter to the Editor: The Association Between Glycemic Variability and the Risk of Diabetic Retinopathy Progression: A Community‐Based Retrospective Cohort Study,” Journal of Diabetes 18, no. 7 (2026): e70250, 10.1111/1753-0407.70250.PMC1336579542444120

Currently the article only lists Wenli Xu as the first author. Yingyan Ma should also have been listed as a first co‐author along with Wenli Xu.

We apologize for this error.

